# Upper urinary dilatation and treatment of 26 patients with diabetes insipidus: A single-center retrospective study

**DOI:** 10.3389/fendo.2022.941453

**Published:** 2022-07-22

**Authors:** Xuesheng Wang, Xiaoqian Ying, Fan Zhang, Xing Li, Guoqing Chen, Zhonghan Zhou, Limin Liao

**Affiliations:** ^1^ Department of Urology, China Rehabilitation Research Center, School of Rehabilitation of Capital Medical University, Beijing, China; ^2^ University of Health and Rehabilitation Sciences, Qingdao, China; ^3^ Cheeloo College of Medicine, Shandong University, Jinan, China

**Keywords:** diabetes insipidus, bladder distension, upper urinary tract dilatation, neurogenic bladder, individualized therapy

## Abstract

**Objective:**

To describe the urinary tract characteristics of diabetes insipidus (DI) patients with upper urinary tract dilatation (UUTD) using the video-urodynamic recordings (VUDS), UUTD and all urinary tract dysfunction (AUTD) systems, and to summarize the experience in the treatment of DI with UUTD.

**Methods:**

This retrospective study analyzed clinical data from 26 patients with DI, including micturition diary, water deprivation tests, imaging data and management. The UUTD and AUTD systems were used to evaluate the urinary tract characteristics. All patients were required to undergo VUDS, neurophysiologic tests to confirm the presence of neurogenic bladder (NB).

**Results:**

VUDS showed that the mean values for bladder capacity and bladder compliance were 575.0 ± 135.1 ml and 51.5 ± 33.6 cmH_2_O in DI patients, and 42.3% (11/26) had a post-void residual >100 ml. NB was present in 6 (23.1%) of 26 DI patients with UUTD, and enterocystoplasty was recommended for two patients with poor bladder capacity, compliance and renal impairment. For the 24 remaining patients, medication combined with individualized and appropriate bladder management, including intermittent catheterization, indwelling catheter and regular voiding, achieved satisfactory results. High serum creatinine decreased from 248.0 ± 115.8 μmoI/L to 177.4 ± 92.8 μmoI/L in 12 patients from a population with a median of 108.1 μmoI/L (IQR: 79.9-206.5 μmoI/L). Forty-four dilated ureters showed significant improvement in the UUTD grade, and the median grade of 52 UUTD ureters decreased from 3 to 2.

**Conclusion:**

Bladder distension, trabeculation and decreased or absent sensations were common features for DI patients with UUTD. Individualized therapy by medication combined with appropriate bladder management can improve UUTD and renal function in DI patients.

## Introduction

Diabetes insipidus (DI) refers to a disease characterized by excretion of abnormally large amounts of dilute urine, which is mainly manifested by hypotonic polyuria (urine output >4 mL/kg/h) and polydipsia (water intake >2 L/m^2^/d) (1). This syndrome can be caused by two fundamentally different mechanisms: inadequate secretion of antidiuretic hormone (ADH) [central diabetes insipidus (CDI)]; or renal insensitivity to ADH [nephrogenic diabetes insipidus (NDI)] (2).

Little is known about the association between DI and urologic complications, bilateral upper urinary tract dilation (UUTD), and severe hydronephrosis without obstruction (3). Patients with potential manifestations of DI, including frequent micturition, urinary tract infection (UTI) and hydroureteronephrosis, may be easily misdiagnosed as neurogenic bladder (NB) (4).

Most studies focusing on DI with hydroureteronephrosis have only been reported in the form of case reports, thus a systematic analysis of clinical characteristics and treatments for DI with UUTD are lacking. Another problem is the lack of a quantitative evaluation of the overall dilation, including hydronephrosis and ureteral dilation, from a coronal and transverse panel magnetic resonance urography (MRU) or 360-degree rotation. Hence, the purpose of this study was to summarize clinical characteristics, assessment, diagnosis, and treatments of patients with DI and UUTD to help urologists better understand this disease and improve the therapeutic effect of DI with UUTD.

## Patients and methods

### Study cohort

**Figure 1:**
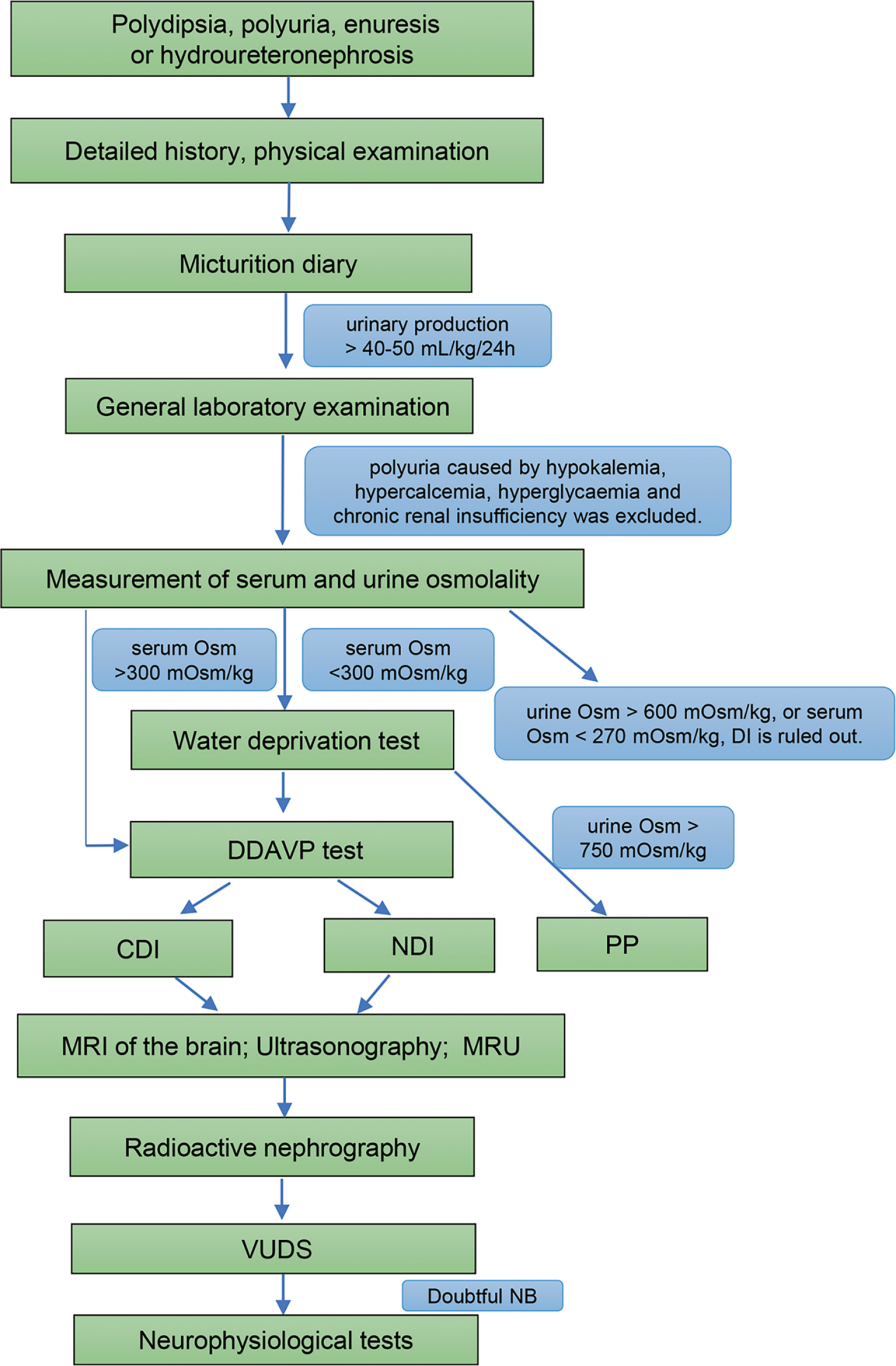
Diagnostic flowchart for DI and DI with NB. Osm, osmolality; DDAVP test, desamino-D-arginine vasopressin test; DI, diabetes insipidus; CDI, central diabetes insipidus; NDI, nephrogenic diabetes insipidus; PP, primary polydipsia; MRI, magnetic resonance imaging; MRU, magnetic resonance urography; VUDS, video-urodynamic recordings; NB, neurogenic bladder.

With the Ethics Committee of the China Rehabilitation Research Centre (No.2021-092-1) approval, we retrospectively reviewed the medical records of 26 patients diagnosed DI with hydroureteronephrosis at our center from January 2010. All of the patients provided a detailed history, physical examination, laboratory testing, the water deprivation test, magnetic resonance imaging (MRI) of the brain, ultrasound of the uropoietic system, MRU, radioactive nephrography, and video-urodynamics (VUDS) before treatment. Moreover, a neurologic examination and neurophysiologic testing were performed in patients with suspected DI and NB (**Figure 1**).

The diagnostic criteria for DI are as follows (5, 6): (1) polydipsia and polyuria, and urinary production > 40-50 mL/kg/24 h in adults; (2) urine specific gravity < 1.005, urinary osmolality < 300 mOsm/kg, and serum osmolality > 300 mOsm/kg; and (3) water deprivation test (+), thus distinguishing CDI from NDI. Polyuria caused by hypokalemia, hypercalcemia, hyperglycemia, chronic renal insufficiency, and primary polydipsia (PP) were excluded.

### Assessment

The micturition diary, a relatively objective detection method, has the advantages of non-invasiveness and repeatability. Moreover, it can identify whether frequent micturition is caused by an increase in absolute urine volume or just the increase in frequency.

The water deprivation test and desamino-D-arginine vasopressin (DDAVP) test, when performed by an experienced physician are helpful to distinguish CDI, NDI, and PP (**Table S1**). The two procedures were both performed as previously described (5, 7).

Sellar tumors, craniocerebral surgery, and craniocerebral trauma account for 17% - 28%, 8% - 49%, and 2% - 11% of all causes of CDI, respectively (8–10). Multiple studies have confirmed that sellar tumors are the most common cause of CDI (8, 9). Therefore, MRI is helpful to recognize the cause of CDI in patients.

The new UUTD and all urinary tract dysfunction (AUTD) grading system, established by Liao, is divided into 5 grades according to the MRU coronal and transverse image panels, and the maximum intensity projection MRU (11, 12). UUTD and AUTD systems can objectively and effectively assess variation tendency in UUTD grades, renal and bladder function, and provide objective indicators for conditions of upper and lower urinary tract function.

Lower urinary tract changes were evaluated using VUDS to assess bladder function. VUDS, consisting of cystometry and simultaneous cystography, was performed on patients based on the International Continence Society (ICS) standards (13).

In a subset of patients with DI, abnormal urodynamics is similar to patients with NB, and neurophysiologic tests are effective ways to diagnose NB. These tests involving sacral reflex, somatosensory evoked potentials (SEPs) and motor evoked potentials (MEPs) provide additional evidence in the search for neurogenic causes (14).

### Management

All patients were classified as CDI or NDI. DDAVP is the primary treatment option for CDI. Unlike CDI treatment, NDI is treated with thiazide diuretics to reduce delivery of the filtrate to nephrons. Based on the VUDS, MRU, and uro-neurophysiologic evaluation, two patients underwent augmentation cystoplasty (AC) due to poor bladder capacity and compliance caused by a NB. Eight patients utilized the intermittent catheterization (IC) regimen for adequate bladder drainage due to detrusor underactivity or acontractile detrusor. Three patients preferred an indwelling catheter over intermittent catheterization to relieve urinary pressure and dilation. The remaining patients were treated with oral medications and a regular self-voiding regimen to alleviate polyuria and UUTD. The DDAVP combined with alpha blocker approach was used for two patients with dysuria and a small PVR. The various therapeutic regimens are summarized in **Table 1**.

**Table 1:** The summary of UUTD and AUTD system, uro-neurophysiological evaluation and therapeutic regimens in DI patients with UUTD.

NO.- diagnosis	Lower Urinary Tract			Upper Urinary Tract		UUTD-degree		Uro-neurophysiological Evaluation		Management
	Detrusor contractility	Bladder sensation	MBC(ml),BC(ml/cmH_2_O),Qmax(ml/s),PVR(ml)	Ureteral obstruction	Renalfunction	Pre-therapy	Post-therapy	Sacral reflex	evoked potentials(MEP/SEP)	
1-DI	Underactive	Reduced	650/32/20/0	B-Y	B-Decompensation	B-3	B-2	Normal	Normal	IC/self-voiding
2-DI	Underactive	Reduced	690/36/19/0	B-N	B- Normal	L-4; R-3	B-2	–	–	IC/self-voiding
3-DI	Underactive	Normal	490/50/13/450	B-N	B- Normal	B-3	B-2	–	–	IC
4-DI	Underactive	Reduced	730/40/9/100	B-Y	B- Compensatory	B-4	L-3; R-2	Normal	Normal	IC
5-DI	Underactive	Normal	630/33/6/450	B-N	B- Normal	L-4; R-3	B-3	–	–	IC
6-DI	Normal	Reduced	730/34/27/20	L-N; R-Y	B- Normal	L-2; R-3	L-1; R-2	–	–	Self-voiding
7-DI	Underactive	Reduced	650/45/20/170	B-Y	B- Normal	B-3	B-2	Normal	Normal	α-blocker+self-voiding
8-DI with NB	Acontractile	Absent	220/9/7/40	B-Y	B-Decompensation	B-4	B-2	Abnormal	Abnormal	AC+IC
9-DI	Normal	Reduced	650/50/31/0	B-N	B- Normal	B-3	L-3; R-2	–	–	Self-voiding
10-DI with NB	Underactive	Normal	500/27/15/170	B-N	B-Decompensation	B-4	B-2	Abnormal	Abnormal	Indwelling catheter
11-DI	Normal	Reduced	550/125/27/10	B-N	B- Normal	L-2; R-3	B-2	–	–	Self-voiding
12-DI with NB	Underactive	Reduced	610/100/15/190	B-N	B-Compensatory	B-4	L-3;R-4	Normal	Abnormal	Indwelling catheter
13-DI	Underactive	Reduced	660/145/23/70	B-N	B- Normal	L-3; R-2	L-2; R-1	Normal	Normal	α-blocker+self-voiding
14-DI	Normal	Reduced	590/45/29/0	B-Y	L-Normal; R-Compensatory	L-2; R-3	B-2	–	–	Self-voiding
15-DI with NB	Acontractile	Absent	530/50/0/530	B-Y	R- Normal; L-Decompensation	L-4; R-3	L-3; R-2	Abnormal	Normal	IC
16-DI	Normal	Normal	450/42/38/0	B-N	B- Normal	B-2	B-1	–	–	Self-voiding
17-DI	Normal	Normal	540/36/29/0	B-N	L-Normal; R-Decompensation	L-3; R-4	L-2; R-3	–	–	Self-voiding
18-DI	Normal	Normal	430/42/30/0	B-N	B- Normal	L-2; R-1	L-2; R-0	–	–	Self-voiding
19-DI with NB	Acontractile	Absent	750/37/0/750	B-Y	R-Decompensation	B-4	B-3	Abnormal	Abnormal	IC
20-DI	Normal	Reduced	510/116/39/10	L-Y; R-N	L-Compensatory; R-Normal	L-3; R-2	B-1	–	–	Self-voiding
21-DI	Underactive	Reduced	700/49/15/450	L-N; R-Y	B- Compensatory	R-4; L-3	B-2	–	–	Indwelling catheter
22-DI	Normal	Normal	450/36/39/0	B-N	B- Normal	L-1; R-2	B-1	–	–	Self-voiding
23-DI	Underactive	Normal	700/25/17/370	B-Y	R- Normal; L-Decompensation	B-3	B-2	Normal	Normal	IC
24-DI with NB	Acontractile	Reduced	280/16/0/280	B-N	B-Decompensation	B-4	B-3	Abnormal	Abnormal	AC+IC
25-DI	Normal	Normal	550/78/39/0	B-Y	B- Normal	B-2	L-2; R-1	–	–	Self-voiding
26-DI	Normal	Reduced	710/43/34/0	L-Y; R-N	B- Normal	L-3; R-4	L-2; R-3	–	–	Self-voiding

DI, diabetes insipidus; UUTD, upper urinary tract dilation; AUTD, all urinary tract dilation; MBC, maximum bladder capacity; BC, bladder compliance; Qmax, maximum flow rate; PVR, post-void residual urine; MEP, motion evoked potential; SEP, somatosensory evoked potential; R, right side; L, left side; B, bilateral side; Y, yes; N, no; IC, intermittent catheterization; AC, augmentation cystoplasty; -, reject.

### Statistical analysis

SPSS (version 22.0; SPSS, Inc., Chicago, IL, USA) was used for statistical analyses. Continuous variables are expressed as the mean ± SD or median (IQR). Frequency (percentage) was used to describe the count data. Differences between groups were assessed with Student’s *t*-test or the Wilcoxon signed rank test for quantitative variables. Statistical significance was defined as a P < 0.05.

## Results

There were 26 patients (average age, 29.2 ± 13.3 years) with the main characteristics of DI who presented enuresis, dysuria and UUTD, of whom 6 were diagnosed with DI accompanied with NB. The etiology of NB, including spinal cord injury, spina bifida, and diabetes mellitus, was established through a detailed history and imaging findings in patients with NB, and further confirmed by neurophysiologic tests. 23 (88.5%) had CDI and 3 (11.5%) had NDI, and median duration of DI was 10.0 years (IQR: 4.3-24.3 years). The demographic and baseline characteristics of the primary assessment are listed in **Table 2**.

**Table 2:** The demographic and based characteristics of the primary assessment.

	Mean + SD/N (%)
Age (years)	29.2 ± 13.3
Duration of symptoms (years) median (IQR)	10.0 (4.3-24.3)
Pulse Rate	78.2 ± 9.7
Systolic pressure	123.5 ± 18.7
Diastolic pressure	83.6 ± 17.7
Sex (N,%)	
Female	3 (11.5%)
Male	23(88.5%)
Chief complaint (N,%)	
Polyuria and polydipsia	11(42.3%)
Hydronephrosis	7 (27.0%)
Dysuria	5 (19.2%)
Enuresis	3 (11.5%)
Water deprivation test (N,%)	
CDI	23(88.5%)
NDI	3 (11.5%)
Etiology (N,%)	
CDI	
Vacuole turcica	2 (8.7%)
Meningitis	3 (13.0%)
Craniocerebral trauma	1 (4.3%)
Craniopharyngioma surgery	1 (4.3%)
Cranial malformations	1 (4.3%)
Idiopathic CDI	15(65.2%)
NDI	
Idiopathic NDI	3 (100%)
Neurogenic bladder	
YES	6 (23.1%)
spinal cord injury	2
spina bifida	3
diabetes mellitus	1
NO	20 (76.9%)

DI, diabetes insipidus; CDI, central diabetes insipidus; NDI, nephrogenic diabetes insipidus; SD, standard deviation; IQR, interquartile range.

**Figure 2:**
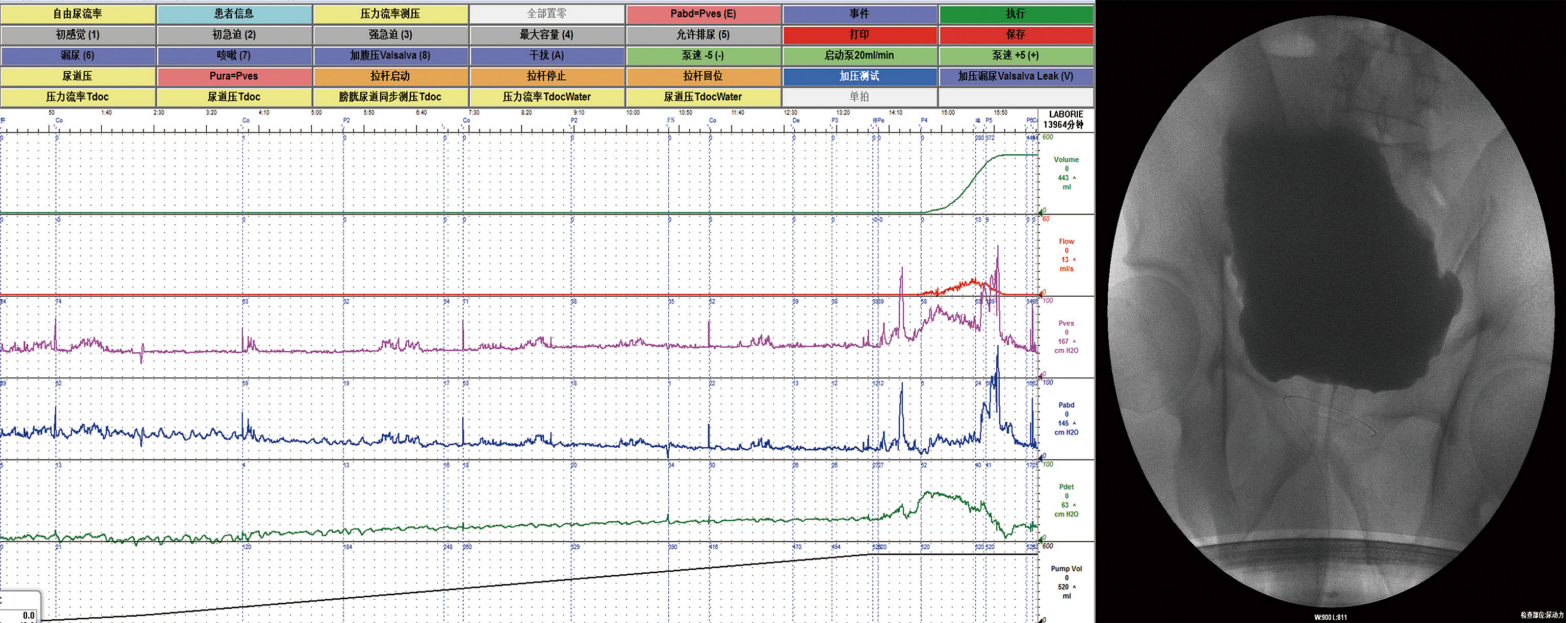
Video-urodynamic recordings revealed high-capacity, impaired sensory function and a trabeculated bladder.

According to the AUTD system, the features of the lower urinary tract in DI patients with UUTD is in **Table 1**. Patients were assessed after admission, revealing detrusor underactivity in 11 patients (42.3%), an acontractile detrusor muscle in 4 patients (15.4%). Interestingly, there were 14 (53.9%) with hyposensitive and 3 (11.5%) with unsensible. High capacity, impaired sensory function, and a trabeculated bladder appeared to be common features in patients with DI (**Figure 2**). VUDS demonstrated a compliant bladder with a large capacity of 575.0 ± 135.1 ml. The average value of bladder compliance was 51.5 ± 33.6 cmH_2_O during the stable filling period. The median PVR and maximum flow rate was 30.0 ml (IQR: 0-302.5 ml) and 20.8 ± 12.1 ml/s in all patients, respectively, and 42.3% of patients (11/26) had a PVR >100 ml (355.5 ± 190.0 ml) due to incomplete bladder emptying.

Clinical follow-up period ranged from 1 to 12 months. The differences in pre- and post-therapeutic parameters for DI patients are shown in **Table 3**. The micturition records showed all patients had improvement in daily water intake and urine volume (6142.3 ± 1680.0 vs. 2850.8 ± 678.1 ml; p<0.01 and 6440.4 ± 1951.7 vs. 2682.2 ± 787.1 ml; p<0.01, respectively). The urinalysis revealed that the specific gravity of urine improved significantly (1.002 ± 0.002 vs. 1.020 ± 0.003; p<0.01), and the urine osmolality was generally elevated (404.3 ± 102.5 vs. 141.8 ± 65.1 mOsm/kg) and the serum osmolality was generally reduced (296.4 ± 19.8 vs. 313.6 ± 22.9 mOsm/kg). According to the AUTD system, the pre-therapeutic serum creatinine (Scr) was > 1.5 mg/dl (1 mg/dl = 88.4 umol/L) in 12 of the patients. The renal function improved in these 12 patients, and the Scr decreased from 248.0 ± 115.8 μmoI/L (pre-therapy) to 177.4 ± 92.8 μmoI/L (post-therapy). The other 14 patients who had a normal Scr (83.8 ± 19.2 μmoI/L) before treatment remained stable. In addition, all patients underwent isotope nephrography to demonstrate single renal function, and outcomes showed 21 kidneys from 13 patients had GFRs < 50 ml/min, and the mean GFRs of those kidneys improved by our treatment (31.2 ± 8.9 vs. 39.4 ± 7.8 ml/min, p<0.05). Although there was no significant improvement in the GFRs of the left and right kidneys from the total population.

**Table 3:** Differences in pre- and post-therapeutic parameters for DI patients.

Characteristics	Pre-therapy	Post-therapy	P-value
Daily fluid intake (ml)	6142.3 ± 1680.0	2850.8 ± 678.1	< 0.01 *
Daily urine output (ml)	6440.4 ± 1951.7	2682.2 ± 787.1	< 0.01 *
Blood urea nitrogen	5.7 ± 2.3	5.8 ± 1.9	0.80
Potassium	3.9 ± 0.5	4.0 ± 0.6	0.79
Serum natrium	147.4 ± 4.5	141.6 ± 4.4	0.63
Serum chloride	110.2 ± 14.9	112.1 ± 14.3	0.44
serum creatinine (μmoI/L) median (IQR)	108.1 (79.9-206.5)	93.9 (76.8-147.5)	< 0.01 *
blood glucose	5.4 ± 1.3	5.0 ± 1.6	0.75
urinary osmolality	141.8 ± 65.1	472.4 ± 105.7	< 0.01 *
serum osmolality	313.6 ± 22.9	294.1 ± 12.5	< 0.01 *
urine specific gravity	1.002 ± 0.002	1.020 ± 0.003	< 0.01 *
Left GFR	49.0 ± 20.7	52.3 ± 19.8	0.43
Right GFR	53.8 ± 17.9	54.5 ± 17.6	0.66
Separation of the left central renal complex	3.8 ± 2.3	2.3 ± 1.7	< 0.01 *
Separation of the right central renal complex	3.7 ± 2.1	2.2 ± 1.5	< 0.01 *

DI, diabetes insipidus; GFR, glomerular filtration rate; IQR, interquartile range; *P < 0.01 versus pre-therapy.

**Figure 3:**
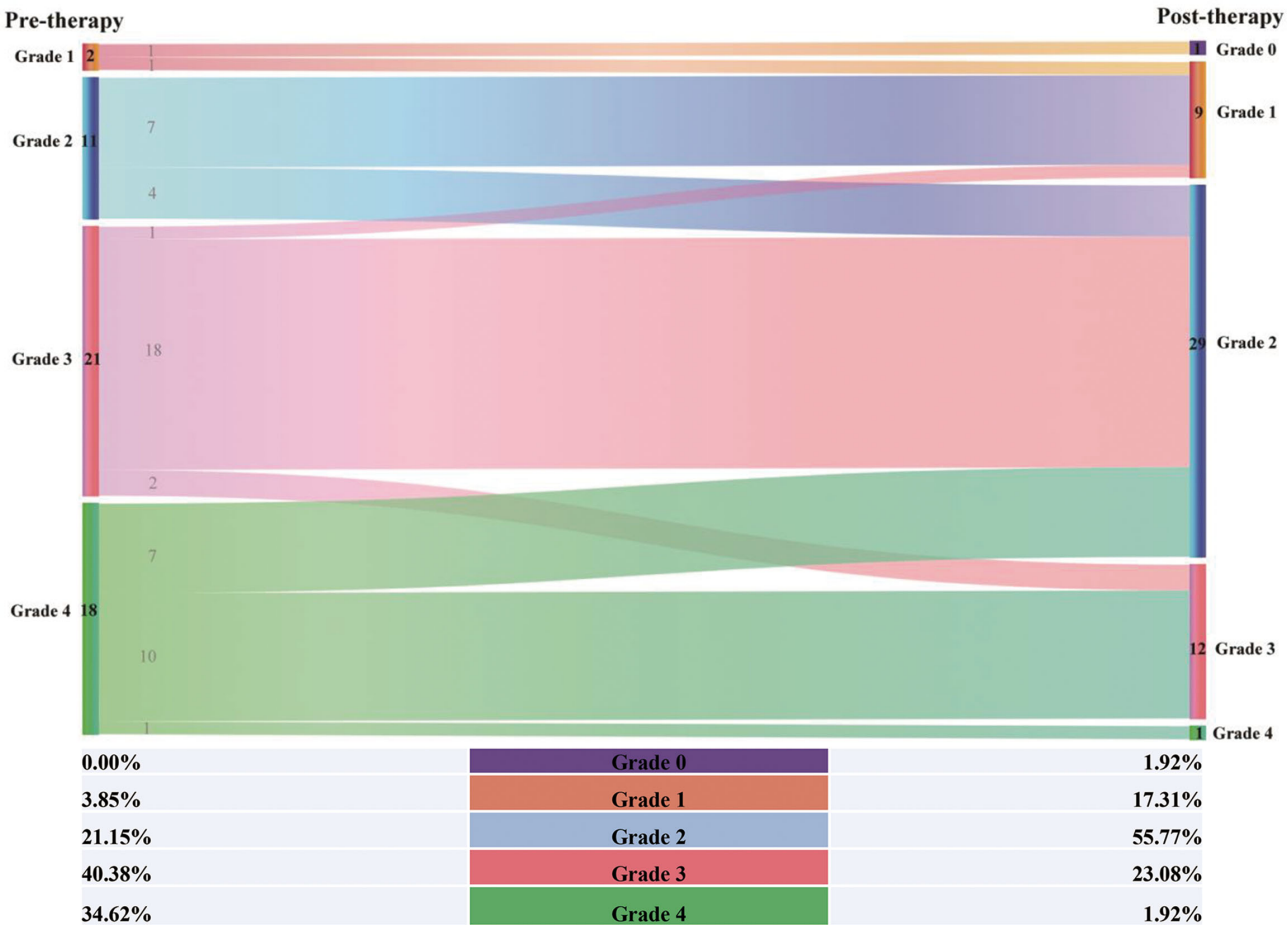
Sankey diagram of grade distribution for pre- and post- therapeutic UUTD.

Ultrasonography showed that there was obvious separation of the central renal complex and dilatation of the ureter in all patients. The separations of the central renal complex for two kidneys improved with proper treatment (left: 3.8 ± 2.3 vs. 2.3 ± 1.7 cm; p<0.01 and right: 3.7 ± 2.1 vs. 2.2 ± 1.5 cm; p<0.01). Indeed, a comprehensive MRU evaluation based on the UUTD and AUTD systems revealed that all patients had bilateral dilations of the pelvis and ureters before treatment (**Supplementary Figure S1**), and the distribution of UUTD in 52 ureters with grades 1-4 was 2, 11, 21, and 18, respectively (**Figure 3**). Combining VUSD with MRU, however, demonstrated that those conditions were not derived from reflux of the pelvis and ureter, and isotope renography and MRU indicated distal ureteral obstruction in 22 upper urinary tract units from 26 patients. The evaluation for urinary tract function using the UUTD and AUTD systems showed a quantitative improvement in the UUTD grades after corresponding therapy (**Figures 3**, **S2**). Forty-four of 52 ureters had significant improvement in dilation, and the median grade of all ureters decreased from 3 to 2 according to the UUTD system (**Figure 3**).

## Discussion

DI, a common disorder with an inability to conserve water and concentrate the urine, frequently results in polydipsia, polyuria, and electrolyte abnormalities (5, 15).

Bladder distension and UUTD are considered rare complications of DI, and prolonged polyuria plays an important role in bilateral, non-obstructive UUTD, which has been reported in patients with DI (3, 16). In our retrospective study 26 DI patients presented with hydronephrosis and dilatation of the ureter when they came to the hospital for evaluation. Polyuria, delayed urination, and distal ureteral obstruction were common features for those patients. Based on these characteristics, we hypothesized that the following factors accounted for the dilatation of the urinary tract in DI: patients with DI produce large urine volumes per unit time, and excessive urine excreted from the kidneys to the ureter can surpass the transport capacity of the urinary tract (17); persistent production of large amounts of urine can lead to bladder distension and hypertrophy, followed by generating intramural obstruction of ureters in the end (3, 18); the duration of detrusor constriction is not long enough to empty the large bladder, which results in a higher PRV (19); and impaired bladder contractility, compromised ureteric peristalsis, and a large PVR deteriorated the dilatation of the urinary tract (20). Moreover, because of individual or social factors, such as study or work, patients cannot urinate when the urge arises, this habit of delaying urination further aggravates UUTD (18, 21).

These above-mentioned characteristics are reflected in urodynamics, which usually present with a high-capacity, compliant bladder and decreased or absent bladder sensations. Our results showed decreased or absent bladder sensations in 17 patients. Furthermore, the investigation of VUDS showed diminished detrusor contractility and a hypertrophic bladder for most DI patients with UUTD (15). Ultrasonography indicated hydronephrosis and ureteral dilatation. However, ultrasound cannot display the kidney and ureter in the same image (11). MRU not only helps urologists understand the condition of the entire urinary tract, but also provides morphologic characteristics for the upper urinary tract and bladder, especially the information on ureteral tortuous knotting and vesicoureteral junction stricture (**Figure S1**). The detailed MRU-UUTD grading and AUTD system proposed by Liao (11, 22) complemented the SFU grading system in the identification of severe (grade III and IV) hydronephrosis. In the present study the UUTD and AUTD systems not only provides the comprehensive evaluation to assess changes in UUTD grades and renal function, but can also be used for making clinical decisions and monitoring UUTD for DI patients. We believed that inflammation in patients with long-term UUTD could result in fibrous band formation or peri-ureteral tissue adhesions. As shown in **Figure S1**, AC with simultaneous ureteroplasty was recommended for patients with grade 3-4 UUTD and renal dysfunction.

Urinary symptoms and imaging findings of most DI patients with UUTD were similar to patients with NB, presenting with urinary frequency, enuresis, dysuria, varying degrees of UUTD, and bladder trabeculation. Nevertheless, many urologists are unfamiliar with this condition and might confuse DI with NB (4). In this study, although none was misdiagnosed as NB, 6 patients with DI were considered to have concomitant NB. Those patients had the definite history of neurologic diseases, and there was abnormal nerve conduction on physical examination. The findings were also confirmed by uro-neurophysiologic abnormalities related to the sacral reflex and evoked potentials. Although NB and DI have similar clinical manifestations, the therapeutic regimens are different for patients, especially in DI patients with NB. Therefore, it is essential to differentiate DI-related diseases and the treatment. It is worth noting that a detailed medical history, physical examination, VUDS, and imaging should be taken into consideration in distinguishing DI with UUTD from NB. Additionally, neurophysiologic tests, providing evidence for searching neurogenic causes, were recommended for NB patients with unknown etiology.

Polyuria is one of the major components of urinary dilatation and renal function failure. Hence, reducing the urine volume and the PVR are critical approaches in the management of DI patients with UUTD (4). Treatment with medication is the first choice to reduce urine output in patients with DI (2). DDAVP is the first-line treatment of CDI, and thiazide diuretics are the most common therapy to ameliorate the polyuria in NDI. Second, we should teach patients the importance of frequent urination on the basis of correct and early diagnosis of the cause of polyuria. On the premise of maintaining this principle, treatment must be individualized in patients with UUTD. Surgical procedures, such as incision of the bladder neck, are aimed at reducing the PVR and alleviating the UUTD (20, 23). We must keep in mind that surgical methods are only adjuvants to medication. For DI patients with NB, an indwelling catheter or CIC must be carried out if retention is prominent. AC was performed on two DI patients with poor bladder capacity, decreased compliance (< 10 ml/cmH_2_O), UUTD (grade≥ III) and renal function dysfunction (Scr≥1.5mg/dL) caused by NB, who achieved satisfactory outcomes. Therefore, we are of the opinion that AC is a feasible option to alleviate progressive renal decline for patients with worsening compliance and deteriorating renal function. In our study, a subset of patients with detrusor contraction but dysuria self-voided with a combination of α-blockers. We managed all patients with individualized treatments to alleviate UUTD and renal deterioration and improve independence and QoL.

At present, there is no evidence on how long DI will develop into hydronephrosis or whether it can be reversed (24); however, even though urinary tract dilation persists for many years, it may also improve after adequate therapy (25). Optimal therapy can result in marked improvement in urinary tract dilatation, even after a short period of time. This conclusion is also consistent with our results that all cases have improved prominently within 1 month after treatment.

Our study had some limitations, including the retrospective design and the small number of patients. In addition, although a summary of our experience can help urologists understand DI with UUTD, the current study was limited by the short follow-up time. Furthermore, because of the limited sample size, further multicenter, clinical trials with a larger sample size are required.

## Conclusion

In conclusion, DI should be considered in patients with polydipsia, polyuria, and urinary tract dilation. Bladder distension, trabeculation, decreased or absent bladder sensations and contractility were common features for DI patients with UUTD Urologists should pay attention to the similarity of symptoms between DI with UUTD and NB, physical and neurological examination, VUDS, neurophysiologic tests were recommended for DI patients with UUTD to differentiate between DI and NB. In DI patients with UUTD, pharmacological treatments reducing fluid intake and urine volume combined with appropriate bladder management can improve UUTD and renal function in DI patients.

## Data Availability Statement

The original contributions presented in the study are included in the article/**Supplementary Material**. Further inquiries can be directed to the corresponding author.

## Ethics Statement

This study protocol was in accordance with the ethical code of the 1975 Declaration of Helsinki and was approved by the Ethics Committee of the China Rehabilitation Research Centre (No.2021-092-1).

## Author Contributions

LL: Project development, Data management, Study coordination. XW: Data management and analysis, Manuscript writing. XY: Data management and analysis. FZ, XL and GC: Data management. ZZ: Data analysis. All authors contributed to the article and approved the submitted version.

## Funding

This study was supported by grants from the National Natural Science Foundation of China (No: 81870523).

## Conflict of Interest

The authors declare that the research was conducted in the absence of any commercial or financial relationships that could be construed as a potential conflict of interest.

## Publisher’s Note

All claims expressed in this article are solely those of the authors and do not necessarily represent those of their affiliated organizations, or those of the publisher, the editors and the reviewers. Any product that may be evaluated in this article, or claim that may be made by its manufacturer, is not guaranteed or endorsed by the publisher.

## Supplementary material

Supplementary Figure 1 MRU-UUTD grades 1–4. **(A)**: Grade 1, slight separation of the central renal complex and the ureter diameter < 7 mm; **(B)**: Grade 2, further separation of the central renal complex and a ureter diameter < 10 mm, a single or a few calices can be visualized; **(C)**: Grade 3, the dilation of renal pelvis, fluid-filled calices pervaded the kidney, the renal parenchyma loss < 50%, and the ureter is tortuous and < 15 mm in diameter; **(D)**: Grade 4, renal pelvis is severely dilated, renal parenchyma loss > 50%, and the ureter is severely tortuous and > 15 mm in diameter. The arrows indicate ureteral stenosis and tortuosity.

Supplementary Figure 2 Comparison of pre- and post-therapeutic MRU in patients based on the UUTD system. A→B and C→D: Hydronephrosis and ureteral dilatation had significant improvement after treatment.
