# Supplementary figures and images for: Upper urinary dilatation and treatment of 26 patients with diabetes insipidus: A single-center retrospective study
Source: Front Endocrinol (Lausanne). 2022 Jul 22;13:941453. doi: 10.3389/fendo.2022.941453 (PMC9354454; doi:10.3389/fendo.2022.941453)

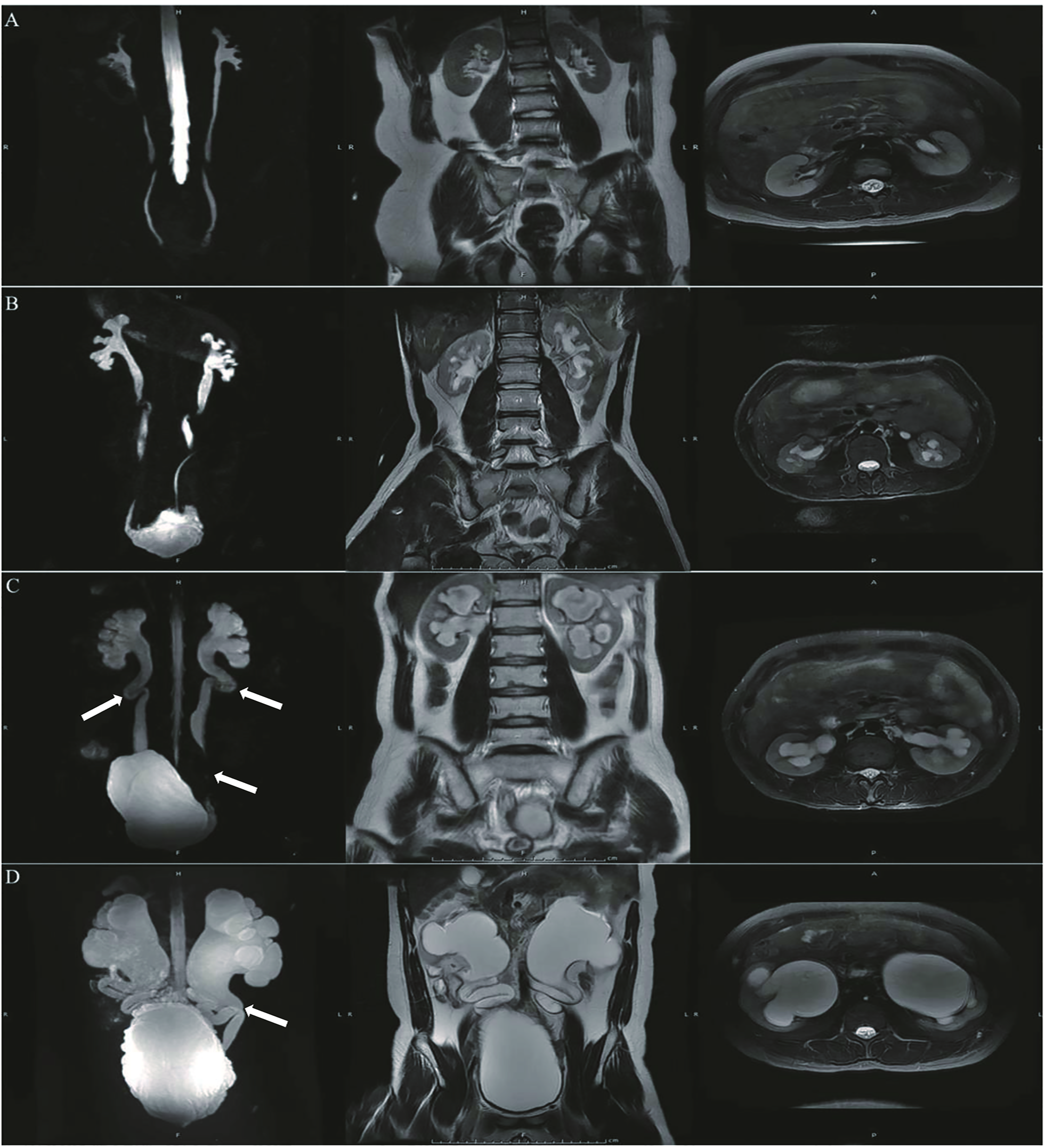

Supplement: Supplementary Figure 1 — MRU-UUTD grades 1–4. (A): Grade 1, slight separation of the central renal complex and the ureter diameter < 7 mm; (B): Grade 2, further separation of the central renal complex and a ureter diameter < 10 mm, a single or a few calices can be visualized; (C): Grade 3, the dilation of renal pelvis, fluid-filled calices pervaded the kidney, the renal parenchyma loss < 50%, and the ureter is tortuous and < 15 mm in diameter; (D): Grade 4, renal pelvis is severely dilated, renal parenchyma loss > 50%, and the ureter is severely tortuous and > 15 mm in diameter. The arrows indicate ureteral stenosis and tortuosity. [file Image_1.tif]

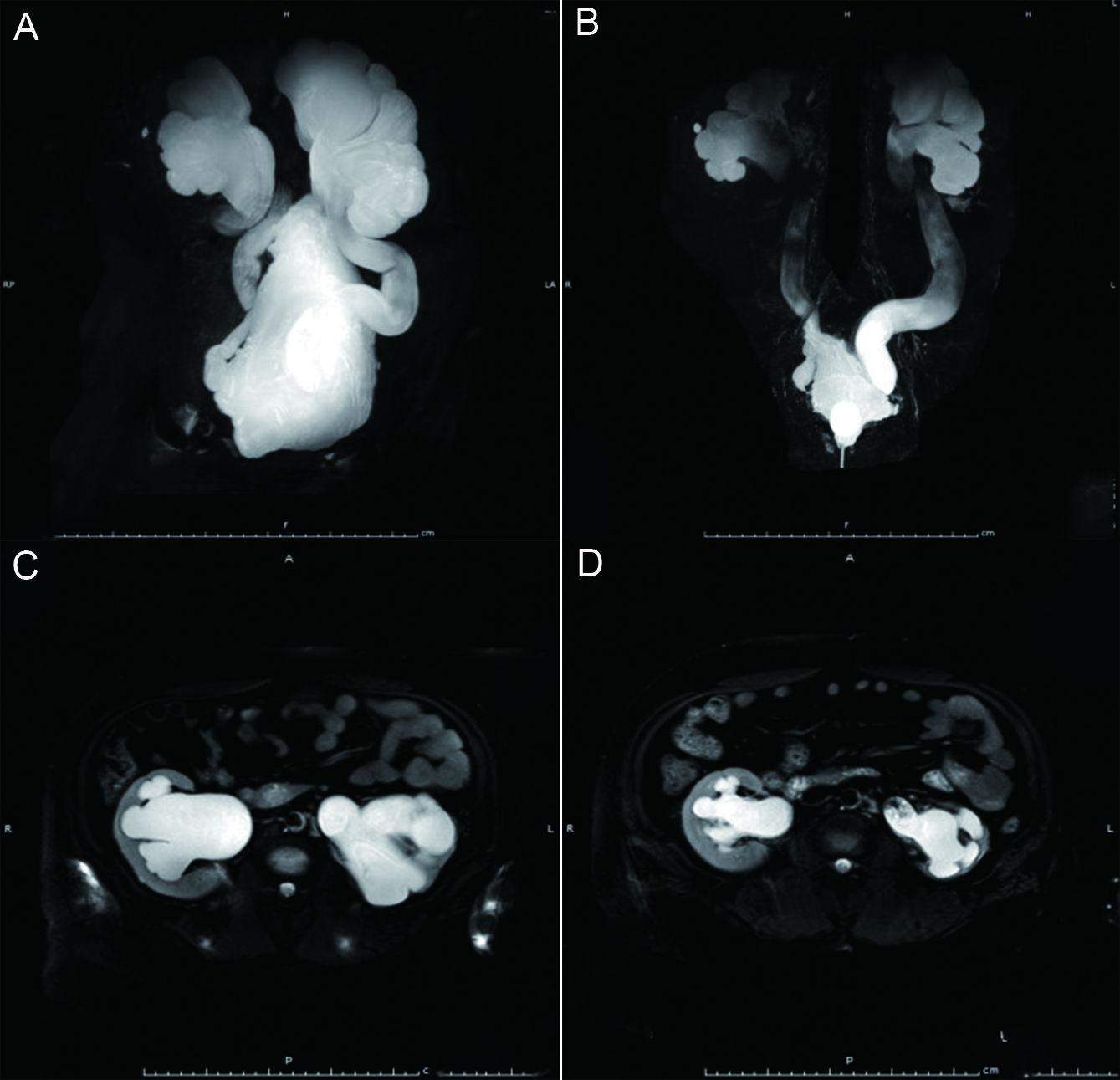

Supplement: Supplementary Figure 2 — Comparison of pre- and post-therapeutic MRU in patients based on the UUTD system. A→B and C→D: Hydronephrosis and ureteral dilatation had significant improvement after treatment. [file Image_2.tif]
