# Supplementary material for: Upper urinary dilatation and treatment of 26 patients with diabetes insipidus: A single-center retrospective study
Source: Front Endocrinol (Lausanne). 2022 Jul 22;13:941453. doi: 10.3389/fendo.2022.941453 (PMC9354454; doi:10.3389/fendo.2022.941453)
Supplement: Supplementary file 3 [file Table_1.docx]

Table S1: The result analysis of the water deprivation test and DDAVP test

| Diagnosis | | Urine osmolality (mOsm/kg) | |
| --- | --- | --- | --- |
|  | | after water deprivation | after DDAVP test |
| CDI | complete DI | <300 | >750 |
|  | partial DI | 300-750 | <750 |
| NDI | | <300 | <300 |
| PP | | >750 | - |

DI: diabetes insipidus; CDI: central diabetes insipidus; NDI: nephrogenic diabetes insipidus; PP: primary polydipsia; DDAVP test: Desamino-D-arginine vasopressin test;
